# Pregnancy Serum DLK1 Concentrations Are Associated With Indices of Insulin Resistance and Secretion

**DOI:** 10.1210/clinem/dgab123

**Published:** 2021-02-26

**Authors:** Clive J Petry, Keith A Burling, Peter Barker, Ieuan A Hughes, Ken K Ong, David B Dunger

**Affiliations:** 1 Department of Paediatrics, Cambridge Biomedical Campus, Cambridge, UK; 2 NIHR Biomedical Research Centre Core Biochemistry Assay Lab, University of Cambridge, Addenbrooke’s Hospital, Cambridge, UK; 3 MRC Epidemiology Unit, Cambridge Biomedical Campus, Cambridge, UK; 4 Institute of Metabolic Science, Cambridge Biomedical Campus, Cambridge, UK

**Keywords:** PREF1, FA1, pregnancy, fetal growth, imprinted

## Abstract

**Context:**

Delta like noncanonical notch ligand 1 (*DLK1*) is a paternally expressed imprinted gene that encodes an epidermal growth factor repeat-containing transmembrane protein. A bioactive, truncated DLK1 protein is present in the circulation and has roles in development and metabolism.

**Objective:**

We sought to investigate links between maternal pregnancy circulating DLK1 concentrations and: (1) maternal and fetal *DLK1* genotypes, (2) maternal insulin resistance and secretion, and (3) offspring size at birth.

**Patients, design, and setting:**

We measured third-trimester maternal serum DLK1 concentrations and examined their associations with parentally transmitted fetal and maternal *DLK1* genotypes, indices of maternal insulin resistance and secretion derived from 75-g oral glucose tolerance tests performed around week 28 of pregnancy, and offspring size at birth in 613 pregnancies from the Cambridge Baby Growth Study.

**Results:**

Maternal DLK1 concentrations were associated with the paternally transmitted fetal *DLK1* rs12147008 allele (*P* = 7.8 × 10^-3^) but not with maternal rs12147008 genotype (*P* = 0.4). Maternal DLK1 concentrations were positively associated with maternal prepregnancy body mass index (*P* = 3.5 × 10^-6^), and (after adjustment for maternal body mass index) with both maternal fasting insulin resistance (Homeostatic Model Assessment of Insulin Resistance: *P* = 0.01) and measures of maternal insulin secretion in response to oral glucose (insulinogenic index: *P* = 1.2 × 10^-3^; insulin disposition index: *P* = 0.049). Further positive associations were found with offspring weight (*P* = 0.02) and head circumference at birth (*P* = 0.04).

**Conclusion:**

These results are consistent with a partial paternal or placental origin for the maternal circulating DLK1 which may lead to increased maternal circulating DLK1 concentrations, stimulation of maternal insulin resistance and compensatory hyperinsulinemia during pregnancy, and the promotion of fetal growth.

The imprinted (paternally expressed) delta like noncanonical notch ligand 1 (*DLK1*) gene encodes an epidermal growth factor repeat-containing transmembrane protein, which has been suggested to have an important role in regulating fetal cell differentiation (1, 2). In addition, recent data indicate that the soluble, truncated form of the protein may be critical for appropriate maternal metabolic adaptation during pregnancy (3). Thus, *DLK1* gene expression may be crucial to our understanding of the imprinted gene regulation of maternal/fetal metabolic interactions during pregnancy.

In the fetus, the imprinted, paternally expressed allele of *DLK1* has been linked to growth (3), and polymorphisms in the gene have been associated with birth weight (4, 5). Hypomethylation of the *DLK1/MEG3* region results in Temple syndrome characterized by fetal growth restriction and shares many of the features of Silver Russell syndrome, including the downregulation of *IGF2* expression (6), another paternally expressed imprinted gene. These observations parallel those in mouse models in which *dlk1* expression is involved in fetal growth (3) and is linked to a wider network of imprinted genes that are vital to fetal development (7).

The truncated DLK1 protein has been detected in the maternal circulation, where it could mediate its potentially critical roles, controlling the adaptation of the maternal metabolism to pregnancy (3). The origin of the protein may be at least partially fetal or placental (8). Circulating DLK1 concentrations rise during the third trimester of pregnancy ((9) (abstract)), reflecting increased fetal weight at that time and reduced circulating concentrations have been associated with fetal growth restriction in humans (3).

In previous studies, we have observed that the expression of other paternally expressed imprinted genes such as *IGF2* can influence maternal metabolism during pregnancy (10-13). In this analysis, we explored the hypothesis that paternally expressed *DLK1* polymorphisms could affect maternal circulating DLK1 concentrations, and that this could affect maternal metabolism and risk of gestational diabetes mellitus (GDM). In turn, this could then affect offspring birth weight and risk of being born small or large for gestational age (SGA/LGA). Confirmation of this sequence of events could have life course significance because *DLK1* gene defects have been associated with increased risk of early puberty, obesity, and type 2 diabetes (14).

To test these hypotheses, we measured third-trimester DLK1 concentrations in serum from mothers who were part of a large pregnancy and birth cohort. We sought associations with parental transmission of *DLK1* polymorphisms, pregnancy outcomes (GDM and gestational hypertension), markers of insulin resistance and secretion derived from an oral glucose tolerance test (OGTT), and offspring size at birth.

## Materials and Methods

### Cambridge Baby Growth Study

The first phase of the prospective, longitudinal Cambridge Baby Growth Study (CBGS) recruited women (and their partners and offspring) attending early pregnancy ultrasound clinics at the Rosie Maternity Hospital, Cambridge, UK, between the years 2001 and 2009 (15). At a median (interquartile range [IQR]) of 28.4 (28.1-28.7) weeks’ gestation 1074 of these women underwent a 75-g OGTT after fasting overnight (11). Venous blood was collected just before and 60 minutes after the glucose load was administered for the measurement of plasma glucose, insulin, and C-peptide concentrations. The fasting blood sample was also used for the measurement of serum DLK1 concentrations. Maternal blood samples for DNA extraction were collected during the OGTT, and from the father and the offspring after birth. After processing the resulting serum samples were stored at -80°C before analyses. Blood pressure measurements during pregnancy were obtained from hospital records and were grouped according to 1 of 4 time points: around weeks 12, 31, 37, and 39 of pregnancy (13).

Birth weights of the offspring were obtained from hospital records. Length (to the nearest 0.1 cm using a SECA 416 Infantometer), head circumferences, and skinfold thicknesses (flank, quadriceps, subscapular, and triceps on the left-hand side of the body using a Holtain Tanner/Whitehouse Calliper; Holtain Ltd., Crymych, UK) were measured in triplicate by 1 of 3 trained pediatric research nurses as soon as possible after birth, at a median (IQR) age of 2 (1-16) days (15). In this cohort, 96.9% of the offspring were White, 0.8% were mixed race, 0.6% were Black (African or Caribbean), 0.8% were East Asian, and 0.9% were South Asian.

### Ethics

The CBGS was granted ethical approval by the Cambridge Local Research Ethics Committee, Addenbrooke’s Hospital, Cambridge, UK (00/325). All procedures followed were in accordance with the institutional guidelines. Written informed consent was obtained from all women and their partners.

### Assays

Serum DLK1 concentrations were measured using an in-house electrochemoluminescent immunoassay (using Meso Scale Discovery reagents, Rockville, MD, USA), developed using Human Pref-1/DLK1/FA1 DuoSet antibodies (R & D Systems [BioTechne], Abingdon, Oxon, UK). The working range of the assay was 0.15 to 40 ng/mL, and inter- and intra-assay imprecision (coefficient of variations) were less than 5% throughout. All other assays were run according to the kit manufacturer’s instructions. Glucose concentrations were measured using a routine glucose oxidase-based method. Plasma insulin concentrations were measured by enzyme-linked immunosorbent assay (Dako U.K. Ltd., Ely, Cambs, UK). The intra-assay coefficient of variation was 4.3% at 82 pmol/L, 3.0% at 402 pmol/L, and 5.7% at 907 pmol/L. Equivalent interassay imprecision was 4.3%, 5.1%, and 5.4%, respectively. C-peptide concentrations were also measured by enzyme-linked immunosorbent assay (DSL Laboratories, London, UK). Intra-assay imprecision was 2.8% at 0.43 nmol/L and 1.47 nmol/L, and 3.2% at 2.80 nmol/L. Equivalent interassay imprecision was 15.7%, 7.8%, and 10.3%, respectively.

### Genotyping

*DLK1* haplotype tag single-nucleotide polymorphisms (SNPs; covering the gene and 20 kb either side of it) were identified by Tagger (using *r*^2^ > 0.8 and minor allele frequency >0.2 as the defining criteria) from the Centre d’Etude du Polymorphisme Humain population of HapMap Project Build 36 using Haploview (16). Genomic DNA was extracted from blood samples or mouth swabs using an Autopure LS Machine (Qiagen Ltd., Crawley, UK). Mother-father-offspring DNA trio samples were genotyped at 5 tag SNPs (rs12147008, rs7155375, rs10139403, rs1802710, rs7147586) using Kompetitive Allele Specific polymerase chain reaction assays, competitive allele-specific polymerase chain reaction SNP genotyping assays that use fluorescence resonance energy transfer quencher cassette oligonucleotides (designed and performed by LGC Genomics, Hoddesdon, UK). All genotypes were consistent with Hardy Weinberg equilibrium (*P* > 0.05 using the χ  ^2^ test) and had a repeat genotyping discordancy rate of <1.0% in 100 samples. Parental transmission of *DLK1* alleles were calculated as previously described (11, 13).

### Definitions of adverse conditions of pregnancy

Evidence of gestational hypertension was sought from the mothers’ hospital notes (defined using the inclusion of a diagnosis of the following terms: “pre-eclampsia,” “gestational hypertension,” or “pregnancy-induced hypertension”). Women were also categorized as having gestational hypertension using the National Institute for Health and Care Excellence criteria (≥140 mm Hg systolic or 90 mm Hg diastolic blood pressure in the second half of pregnancy in women without chronic hypertension) (17), with the exception that for our analysis evidence of gestational hypertension was accepted if the blood pressure cutoffs were exceeded at 1 reading rather than at least 2 (13). The International Association of Diabetes in Pregnancy Study Groups’ thresholds for 0- and 60-minute OGTT glucose concentrations (ie, >5.1 and 10.0 mmol/L, respectively (18)) were used as the criteria to define the presence of GDM. The 120 minute-plasma glucose concentrations were only measured from May 2007 onwards, so were not used in this analysis to define GDM (only 7% of UK women with GDM receive a diagnosis based solely on the 120-minute measurement (19)). SGA was defined as birth weight for gestational age of greater than 1.5 (internal) SDs lower than the mean and LGA as greater than 1.5 SDs higher than the mean (20).

### Calculations

Body mass index (BMI) was calculated as the weight (prepregnancy for the mother and shortly after birth for the baby) divided by the height (or length) squared. Skinfold thickness measurements were analyzed as means of 3 readings and are presented as the mean skinfold thickness of the 4 sites combined. Homeostatic Model Assessment (HOMA) modelling, which has been validated in pregnancy (21), was performed using fasting circulating glucose and C-peptide concentrations and the online HOMA calculator (available at https://www.dtu.ox.ac.uk/homacalculator/) (22). Indices of insulin secretion were calculated using insulin concentrations. The insulin increment was calculated as the OGTT 60-minute insulin concentrations minus the fasting concentrations. The insulinogenic index was calculated as the insulin increment divided by the corresponding glucose increment. The insulin disposition index was calculated as the insulinogenic index multiplied by the fasting insulin concentration.

### Statistics

Inclusion criteria for this analysis were availability of a fasting OGTT serum sample and trio *DLK1* SNP genotypes. In preliminary analyses, twin pregnancies had clearly higher maternal serum DLK1 concentrations, with mean (95% CI) ranks: 566 (461-670) (n = 11) versus singleton pregnancies: 308 (294-322) (n = 613) (*P* = 2.2 × 10^–6^), so twin pregnancies were excluded from the analysis. This left a total of 613 pregnancies that were included in this analysis. Associations between maternal DLK1 concentrations (the dependent variable in some models and independent in others) and continuous variables were assessed using linear rank regression (23) because the DLK1 concentrations (and residuals in linear statistical models) were positively skewed and remained so even after logarithmic transformation (as assessed using Stata’s “sktest” skewness and kurtosis test for normality). Genetic associations (using parentally transmitted fetal *DLK1* alleles or maternal *DLK1* genotypes, adjusted for maternal BMI) with maternal DLK1 concentration ranks were also tested using linear rank regression. For binary outcomes, logistic rank regression was used. Associations between 2 categorical variables were analyzed using either the χ  ^2^ or Fisher’s exact tests. Other associations not involving DLK1 concentrations were assessed using standard linear or logistic regression, adjusted for confounders as appropriate. Subgroup analyses (testing associations with the maternal indices of insulin resistance and secretion) were performed using groups derived from the overall population according to the paternally transmitted fetal *DLK1* rs12147008 allele. Unless otherwise stated, all other data are presented as means (95% CIs). Linkage disequilibrium between genetic variants was assessed using the R (version 3.6.1) package “genetics” (version 1.3.8.1.2) (24). Other statistical analyses were performed using Stata, version 13.1 (StataCorp LP, College Station, TX, USA). *P* < 0.05 was considered statistically significant throughout, except for the genetic associations in which a Benjamini-Hochberg correction was applied for multiple testing (25), using a false discovery rate of 10%.

## Results

### Study participants

The clinical and demographic traits of the 613 women and babies included in the analysis showed modest differences to those of other CBGS participants (Table 1) (with nongenetic study data available (26)): mothers who were included in the analysis were slightly younger, less likely to smoke, more likely to be nulliparous, had higher weight gains during pregnancy, and gave birth around 1 day later.

**Table 1.** Characteristics of CBGS pregnant women and their babies included in this analysis and those excluded from it

Characteristic	Included in the Analysis	Excluded From the Analysis	*P* Values
Marital status (married/cohabiting/lone parent), n	449/85/15	634/111/13	0.4
Highest qualification (high school/university entrance/ degree or above), n	53/79/236	60/106/256	0.5
Age, y	33.2 (32.9 to 33.6) (n = 561)	33.7 (33.4 to 34.0) (n = 774)	0.045
Prepregnancy BMI, kg/m^2^	24.0 (23.6 to 24.4) (n = 498)	24.1 (23.8 to 24.5) (n = 689)	0.6
Parity, 0/1/2/3/4/5	292/212/75/19/6/2	425/428/140/42/2/4	0.01
Weight gain in pregnancy, kg	9.0 (8.4 to 9.7) (n = 379)	7.6 (7.0 to 8.2) (n = 495)	1.6 × 10^–3^
Smoked during pregnancy, n	Yes 21/no 586	Yes 65/no 983	0.02
Developed GDM	Yes 55/no 558	Yes 56/no 415	0.1
Developed gestational hypertension	Yes 17/no 288	Yes 27/no 388	0.6
Gestational age at baby’s birth, wk	40.0 (39.9 to 40.1) (n = 609)	39.7 (39.6 to 39.8) (n = 1048)	5.7 × 10^–5^
Baby’s birthweight adjusted for gestational age, sex, parity, and maternal BMI, kg	3.50 (3.46 to 3.53) (n = 497)	3.46 (3.43 to 3.49) (n = 685)	0.2

Parentheses enclose the 95% CIs and/or numbers of participants.

Abbreviations: BMI, body mass index; CBGS, Cambridge Baby Growth Study; GDM, gestational diabetes mellitus.

### Maternal serum DLK1 concentrations and maternal characteristics

All serum samples contained detectable concentrations of DLK1, with a median serum DLK1 concentration of 3.0 (IQR, 2.3-3.8; range, 1.1-54.7) ng/mL (n = 613) at 28.4 (IQR, 28.1-28.7) weeks’ gestation. Maternal prepregnancy BMI was positively associated with serum DLK1 concentration ranks at week 28 of pregnancy (*P* = 3.5 × 10^-6^; Table 2). Conversely, weight gains in pregnancy (adjusted for prepregnancy BMI) were negatively associated with serum DLK1 concentration ranks (*P* = 0.04). Maternal serum DLK1 concentration rank was positively associated with risk of gestational hypertension (*P* = 0.01; adjusted for prepregnancy BMI) but not with GDM status (Table 2).

**Table 2.** Maternal serum DLK1 Concentrations, at 28 weeks’ gestation, associated with maternal traits in pregnancy

Maternal Traits	Unadjusted			Adjusted for Prepregnancy BMI		
	β′ or Odds Ratio	*P* Value	N	β′ or Odds Ratio	*P* Value	n
Age, y	-0.008 (-0.089 to 0.074)	0.9	561	-0.040 (-0.126 to 0.049)	0.4	469
Prepregnancy BMI, kg/m^2^	0.206 (0.114 to 0.278)	3.5 × 10^–6^	498	N/A		
Pregnancy weight gain, kg	-0.080 (-0.184 to 0.021)	0.1	379	-0.110 (-0.217 to 0.008)	0.04	374
Parity, n	-0.038 (-0.124 to 0.044)	0.4	606	-0.077 (-0.164 to 0.013)	0.1	498
GDM (yes/no)	1.000 (0.998 to 1.001)	0.6	613	1.000 (0.999 to 1.002)	0.3	498
Gestational hypertension (yes/no)	1.004 (1.001 to 1.007)	9.4 × 10^–3^	305	1.005 (1.001 to 1.008)	0.01	253

Standardized regression coefficients are SDs of the maternal trait per SD of the maternal DLK1 concentration rank. β and odds ratio are presented as means (95% CIs).

Abbreviations: BMI, body mass index; N/A, not available.

### Maternal serum DLK1 concentrations and maternal/fetal DLK1 SNP genotypes

Paternally transmitted fetal *DLK1* rs12147008 alleles were associated with maternal serum DLK1 concentration ranks: C allele 257 (218-296) ranks (n = 75) versus T allele 315 (297-333) ranks (n = 342) (*P* = 7.8 × 10^-3^, adjusted for maternal prepregnancy BMI). However, maternal genotypes of the same SNP were not associated with the DLK1 concentration ranks: C/C 289 (246-333) (n = 29) versus C/T 300 (278-322) (n = 144) versus T/T 311 (291-330) (n = 297) (*P* = 0.4, adjusted as previously). After correction for multiple testing, no associations with other fetal paternally transmitted *DLK1* alleles or maternal *DLK1* genotypes were observed (Table 3).

**Table 3.** Associations between paternally transmitted fetal alleles and maternal genotypes for haplotype tag DLK1 SNPs with maternal serum DLK1 concentration ranks

*DLK1* SNP	Ancestral > Derived Allele	Derived Allele Frequency	Paternally Transmitted Fetal Allele		Maternal Genotype	
			Effect of Derived Allele	*P* Value	Effect of Derived Genotype	*P* Value
rs12147008	C > T	0.77	+	7.8 × 10^–3^	(+)	0.4
rs7155375	C > T	0.36	(-)	0.1	(-)	0.6
rs10139403	A > G	0.68	(+)	0.04	(+)	0.4
rs1802710	T > C	0.49	(-)	0.3	(+)	0.7
rs7147586	C > T	0.29	(-)	0.8	(-)	0.5

Using the Benjamini-Hochberg correction for multiple testing, the only association that reached statistical significance was the one with paternally transmitted fetal rs12147008. All *P* values adjusted for maternal prepregnancy body mass index. Directions of associations are shown in parentheses where the *P* value is >0.01.

### Maternal serum DLK1 concentrations and OGTT indices of insulin resistance and secretion

**Figure 1.**
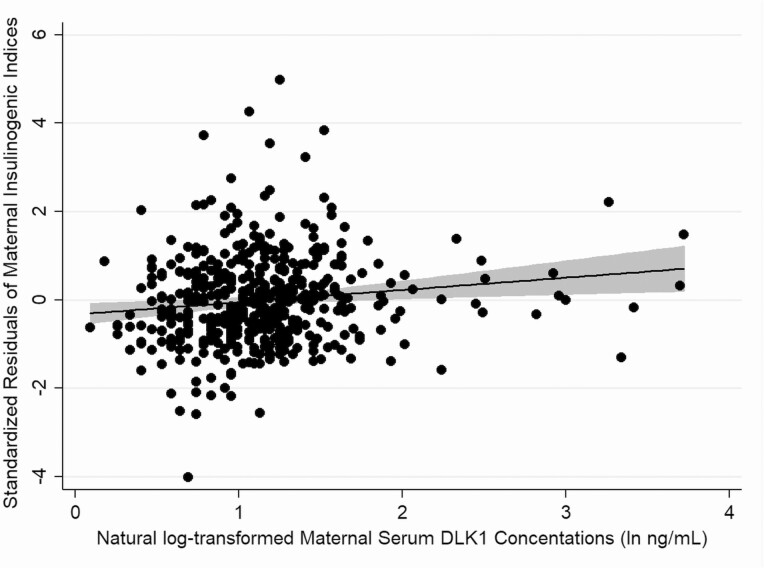
Scatter plot of the standardized residual maternal insulinogenic indices (adjusted for prepregnancy BMIs) by the natural log-transformed serum DLK1 concentrations at week 28 of pregnancy. The line of best fit of the modeled data is shown (in black), along with its associated 95% CI (in gray). Although these data are plotted as is for ease of interpretation, because of significant skewness of the log-transformed DLK1 concentrations and their residuals (both *P* < 0.0001), in the main analysis DLK1 concentration ranks were used instead of the log-transformed concentrations. BMI, body mass index.

Consistent with the lack of association with GDM, maternal serum DLK1 concentrations were not associated with OGTT glucose concentrations (Table 4). Adjusted for maternal BMI, they were however positively associated with OGTT indices of insulin secretion (insulin increment (*P* = 2.3 × 10^-4^), insulinogenic index (*P* = 1.2 × 10^-3^; Fig. 1), and insulin disposition index (*P* = 0.049)). Maternal serum DLK1 concentration ranks were also positively associated with indices of insulin resistance (C-peptide derived HOMA of Insulin Resistance [HOMA IR]: *P* = 0.01, and fasting C-peptide concentration: *P* = 5.6 × 10^-3^).

**Table 4.** Maternal serum DLK1 concentrations, at 28 weeks’ gestation, associated with maternal OGTT indices and analyte concentrations

Maternal Traits	Unadjusted			Adjusted for Prepregnancy BMI		
	β′	*P* Value	N	β′	*P* Value	n
Fasting glucose concentration, mmol/L	-0.010 (-0.074 to 0.058)	0.8	613	0.067 (-0.016 to 0.125)	0.1	498
60-min glucose concentration, mmol/L	-0.048 (-0.113 to 0.017)	0.1	606	0.031 (-0.040 to 0.102)	0.4	493
C-peptide derived HOMA IR, 100/%S	0 (0 to 0.001)	0.9	551	0.117 (0.026 to 0.208)	0.01	447
Fasting C-peptide concentration, nmol/L	0.024 (-0.062 to 0.112)	0.6	551	0.131 (0.039 to 0.224)	5.6 × 10^–3^	447
60-min C-peptide concentration, nmol/L	0.100 (0.014 to 0.187)	0.02	546	0.170 (0.077 to 0.262)	3.3 × 10^–4^	443
Insulin increment, pmol/L	0.091 (0.010 to 0.173)	0.03	602	0.162 (0.076 to 0.248)	2.3 × 10^–4^	488
Insulinogenic index	0.168 (0.086 to 0.250)	6.9 × 10^–5^	574	0.154 (0.061 to 0.246)	1.2 × 10^–3^	467
Insulin disposition index	0.170 (0.085 to 0.255)	9.8 × 10^–5^	574	0.095 (0 to 0.189)	0.049	467

Standardized regression coefficients are SD of the maternal trait per SD of the maternal DLK1 concentration rank. β presented as mean (95% CI).

Abbreviations: BMI, body mass index; HOMA-IR, Homeostatic Model Assessment for Insulin Resistance; OGTT, oral glucose tolerance test.

The subgroup analyses still showed associations between maternal DLK1 concentrations and OGTT indices of insulin secretion (insulin increment [*P* = 0.047 and 0.5 for the major and minor rs12147008 allele subgroups, respectively], insulinogenic index [*P* = 0.003 and 0.01], and insulin disposition index [both *P* = 0.005]) (Table 5). However, there were no significant associations with either C-peptide derived HOMA IR or fasting C-peptide concentration.

**Table 5.** Subgroup analysis of the associations between maternal DLK1 concentrations and indices of insulin resistance and secretion, grouped according to the paternally transmitted fetal DLK1 rs12147008 allele

Index of Insulin Resistance or Secretion	Paternally Transmitted Fetal *DLK1* rs12147008 Allele					
	Major Allele			Minor Allele		
	β′	*P*	n	β′	*P*	n
C-peptide derived HOMA IR	-0.023 (-0.125 to 0.080)	0.7	373	-0.063 (-0.276 to 0.150)	0.6	79
Fasting C-peptide concentration	-0.001 (-0.102 to 0.104)	1.0	373	-0.059 (-0.266 to 0.149)	0.6	79
Insulin increment	0.102 (0.002 to 0.202)	0.047	404	0.085 (-0.148 to 0.318)	0.5	88
Insulinogenic index	0.161 (0.054 to 0.269)	0.003	384	0.281 (0.064 to 0.500)	0.01	85
Insulin disposition index	0.159 (0.049 to 0.269)	0.005	384	0.317 (0.101 to 0.534)	0.005	85

Abbreviations: *DLK1*, delta like noncanonical notch ligand 1; HOMA-IR, Homeostatic Model Assessment for Insulin Resistance.

### Maternal serum DLK1 concentrations and offspring birth characteristics

Positive associations were found between maternal serum DLK1 concentration ranks and offspring weight (*P* = 0.02), head circumference (*P* = 0.04), and BMI (*P* = 0.03) (all adjusted for confounders including gestational age) at birth but not with length or skinfold thickness (Table 6). Accordingly, maternal serum DLK1 concentration ranks were also associated with higher risk of LGA at birth (*P* = 0.04).

**Table 6.** Maternal serum DLK1 concentrations, at 28 weeks’ gestation, associated with offspring size at birth

Neonatal Traits	Unadjusted			Adjusted Models		
	β′ or Odds Ratio	*P* Value	N	β′ or Odds Ratio	*P* Value	n
Weight, kg	0.001 (-0.075 to 0.077)	1.0	607	0.091 (0.016 to 0.166)	0.02	497
Length, m	-0.035 (-0.113 to 0.043)	0.4	581	0.023 (-0.044 to 0.091)	0.5^*a*^	478
Head circumference, m	0.005 (-0.071 to 0.082)	0.9	582	0.067 (0.002 to 0.132)	0.04^*a*^	481
BMI, kg/m^2^	0.017 (-0.063 to 0.097)	0.7	579	0.081 (0.007 to 0.155)	0.03^*a*^	477
Mean skinfold thickness, m	-0.021 (-0.101 to 0.059)	0.6	581	-0.008 (-0.087 to 0.072)	0.8^*a*^	479
SGA, yes/no	0.999 (0.997 to 1.000)	0.2	607	0.998 (0.996 to 1.000)	0.2	497
LGA, yes/no	1.001 (1.000 to 1.003)	0.1	607	1.002 (1.000 to 1.004)	0.04	497

Adjusted models: adjusted for prepregnancy maternal BMI, sex of baby, parity, and gestational age at birth. Standardized regression coefficients are standard deviations of the maternal trait per standard deviation of the maternal DLK1 concentration rank. β and odds ratio are presented as means (95% CIs).

Abbreviations: BMI, body mass index; LGA, large for gestational age; SGA, small for gestational age.

^*a*^Additionally adjusted for neonatal age at measurement.

## Discussion

In this analysis, concentrations of the soluble form of DLK1 in the maternal circulation were found to be associated with a paternally inherited fetal *DLK1* allele but not with maternal genotypes. These circulating DLK1 concentrations were also associated with maternal C-peptide–derived HOMA IR and fasting C-peptide concentrations, as well as with OGTT insulin increments, insulinogenic indices, and the insulin disposition indices, in the third trimester of pregnancy. The data would be consistent with the possibility of DLK1 having a role in the development of insulin resistance of pregnancy combined with stimulating compensatory hyperinsulinemia.

In the present analysis, there was a significant association between maternal DLK1 concentration ranks and a paternally transmitted fetal *DLK1* allele (rs12147008, tested as part of 5 *DLK1* variants chosen to represent 80% of the common variation in the gene plus 20 kb upstream and downstream of it). This SNP displays some degree of linkage disequilibrium with *DLK1* SNPs rs10144381 and rs12881760 (both *r*^2^ = 0.457, D′ = 0.879 in the CEU population [Centre d'Etude du Polymorphisme Humain Utah residents with ancestry from Western Europe]), which have been reported to be associated with blood DLK1 concentrations in the equivalent direction in large genome-wide association studies (27, 28). In our analysis, there were no associations between circulating DLK1 concentrations and the maternal rs12147008 genotypes, so circulating maternal DLK1 is likely to be at least partially of fetal or fetal-derived placental origin. Although paternally transmitted fetal *DLK1* rs12147008 was not significantly associated with any other maternal or offspring phenotypes in our modestly sized analysis (data not shown), it displays modest linkage disequilibrium with rs6575803 (*r*^2^= 0.113, D′ = 0.466) in the CEU population, which was the lead *DLK1* SNP reported to be associated with birth weight in genome-wide association study analyses (4, 5). This observation combined with the association between DLK1 concentrations and indices of insulin secretion in our subgroup analyses support a direct functional effect of DLK1 protein concentrations. However, these results are more difficult to interpret because of the drop in statistical power associated with the reduced number of participants involved.

The associations that we found between maternal serum DLK1 concentrations in pregnancy and markers of insulin resistance (HOMA IR and fasting C-peptide concentrations) have not been reported previously. However, there are some data from men linking DLK1 concentrations to insulin resistance. In a recently published study of 68-year-old men, circulating DLK1 concentrations were positively correlated with insulin resistance measured by euglycemic hyperinsulinemic clamps and HOMA IR, as well as being negatively associated with muscular glucose disposal rates (29). In another study of middle-aged men, serum DLK1 concentrations were also positively associated with insulin resistance as assessed in intravenous glucose tolerance test studies (30). Mouse models with increased circulating dlk1 concentrations exhibit increased growth hormone secretion and igf-i resistance (31), and decreased muscular Glut4 expression (29), which provides a putative mechanism for DLK1-stimulated insulin resistance. Despite its associations with fasting insulin resistance and compensatory hyperinsulinemia, we found no association between circulating DLK1 levels and maternal glycaemia or GDM risk, which is consistent with the findings of Wurst et al (32). Li et al (33) found lower DLK1 concentrations were associated with GDM, but this was in cord blood in only a small number of pregnancies.

In contrast to the null association of DLK1 concentrations with GDM risk in the present analysis, which is compatible with the reported lack of association with hepatic glucose output (29), in a subset of women for whom blood pressure measurements in pregnancy were available, we found that circulating DLK1 concentrations were positively associated with a higher risk for gestational hypertension despite their association with increased offspring birthweight. This is consistent with our previous suggestion that fetal-imprinted genes are able to influence maternal metabolism and physiology in pregnancy (34). The mechanism of the association between circulating DLK1 concentrations and gestational hypertension is unknown, although our data (not shown) suggest that it is independent of insulin resistance. One study, which may be relevant to it, however, found that preeclampsia was associated with increased *DLK1* expression in human umbilical vein endothelial cells, accompanied by lower secretion of nitrite, vascular endothelial growth factor, and higher secretion of endothelin-1 (35).

We also observed positive associations between maternal DLK1 concentrations and offspring birthweight, BMI, and head circumference, and with higher risk for being born LGA. This is consistent with the previously reported link between increased placental *DLK1* expression and higher risk of being LGA (36). We observed no altered risk of SGA in the offspring, unlike a previous study (37). The lack of association in our analysis may relate to the liberal definition of SGA that we used (20), which was chosen because of the small number of SGA pregnancies available to us if we had used a more traditional cutoff of -2 SDs. In addition to the positive associations with birthweight and risk of LGA, we also observed a positive association between maternal DLK1 concentration ranks and offspring BMI at birth. However, there was no apparent association with offspring skinfold thicknesses. This suggests that the increased offspring BMI associated with higher maternal DLK1 concentration ranks may result from increased lean mass as well as adiposity.

In humans, it is still not clear whether the increased maternal circulating DLK1 concentrations observed in late pregnancy (9) are of fetal, placental, or maternal origin, or a combination of these. The strong positive association that we observed between circulating maternal DLK1 concentrations at week 28 of pregnancy and prepregnancy BMI suggests that a proportion of the circulating DLK1 protein may be maternal in origin. The precise source of this circulating DLK1 before pregnancy is currently unknown (29), although it is unlikely to be adipose tissue, which does not appear to express it (38, 39). It has been reported that pregnant women with higher BMIs (and therefore higher circulating DLK1 concentrations) tend to have larger placentas at term (40). In addition, placental weights are reported to be correlated with circulating DLK1 concentrations (37). Because placentas are mainly fetal in origin (41), the association between maternal prepregnancy BMI and pregnancy DLK1 concentrations does not therefore rule out a fetal or placental origin for maternal circulating DLK1. One study that investigated the origin of circulating DLK1 in pregnancy reported 1- to 2-fold higher cord serum DLK1 concentrations than in serum from maternal peripheral blood in late pregnancy (8), suggesting secretion from the fetus or placenta. In another study, ex vivo placentas secreted plentiful amounts of DLK1 protein (37). Although in a study of mouse models in which circulating dlk1 concentrations were genetically manipulated, it was suggested that circulating dlk1 was primarily of fetal origin. It was also suggested that a potential further source of dlk1 was from a specific trophoblast population of cells in the placenta (3). The weight of evidence from humans therefore suggests that maternal circulating DLK1 may be largely of placental origin; our paternally transmitted fetal *DLK1* genotype association is consistent with this.

Our study has a number of limitations. First, insulin resistance and secretion were estimated using surrogate indices rather gold standard techniques such as euglycemic or hyperglycemic hyperinsulinemic clamps. We chose to calculate HOMA IR values using C-peptide concentrations because, unlike insulin, they are unaffected by either hepatic insulin extraction or variations in insulin degrading enzyme activity (42). In contrast, indices of insulin secretion were calculated using insulin concentrations because of its much shorter circulating half-life than that of C-peptide, allowing for greater sensitivity. Despite not being gold standard techniques, the use of HOMA-derived indices (43), including specifically in pregnancy (21), and the oral-derived insulin disposition index (44) have been validated against more labor-intensive techniques, using either gold standard clamps or more sophisticated techniques that have also been validated against clamps (45). A further limitation of our analysis is that, because of unavailability of 30-minute samples, the insulinogenic and disposition indices were calculated using 60-minute insulin concentrations, despite it being the 30-minute insulin concentration that has been validated against first-phase insulin secretion (42). However, OGTT 60-minute insulin concentrations have previously been shown to correlate with the 30-minute concentrations (46), suggesting that they should still be useful in the assessment of insulin secretion. Third, our DLK1 concentrations are cross-sectional and derived from a single timepoint, limiting the degree of putative causality that can be inferred from the associations. Also, placental weights were not available to us. An alternative explanation for our observed associations, although inconsistent with our genetic data, is that first-trimester insulin resistance, leading to hyperinsulinemia, may have resulted in increased placental size (47) and therefore DLK1 secretion. Our cross-sectional observations and lack of placental weights mean that we are unable to test this explanation. Finally, the genetic associations tested are based on a relatively small sample and need to be confirmed in another population. However, our data provide additional evidence consistent with the possibility of maternal circulating DLK1 protein having at least a partial fetal or fetal-derived placental origin.

In summary, the present analysis is the first to investigate possible links between maternal circulating DLK1 and insulin resistance and secretion in the third trimester of pregnancy. Our results are consistent with the hypothesis that human circulating DLK1 is, at least partially, of (fetally derived) placental origin in pregnancy and may have a role in stimulating both maternal insulin resistance and compensatory hyperinsulinemia, which in turn might increase fetal weight gain due to the increased availability of nonglucose nutrients.

## Data Availability

Data from this analysis, with the exception of the *DLK1* genotypes for ethical and consensual reasons, are available at https://doi.org/10.17863/CAM.47262.

## Abbreviations

BMI: body mass index
CBGS: Cambridge Baby Growth Study
DLK1: delta like noncanonical notch ligand 1
GDM: gestational diabetes mellitus
HOMA: Homeostatic Model Assessment
HOMA-IR: Homeostatic Model Assessment for Insulin Resistance
IQR: interquartile range
LGA: large for gestational age
OGTT: oral glucose tolerance test
SGA: small for gestational age
SNP: single-nucleotide polymorphism.

## Additional Information

***Disclosures:*** The authors report no conflicts of interest in this work.
